# Effect of different drying techniques on flowability characteristics and chemical properties of natural carbohydrate-protein Gum from durian fruit seed

**DOI:** 10.1186/1752-153X-7-1

**Published:** 2013-01-04

**Authors:** Hamed Mirhosseini, Bahareh Tabatabaee Amid

**Affiliations:** 1 Department of Food Technology, Faculty of Food Science and Technology, University Putra Malaysia, 43400 UPM, Serdang, Selangor, Malaysia

**Keywords:** Carbohydrate biopolymer, *Durio zibethinus*, Agricultural biomass waste, Solubility, Foaming properties, Water holding capacity, Oil holding capacity, Flow characteristics

## Abstract

**Background:**

A natural carbohydrate biopolymer was extracted from the agricultural biomass waste (durian seed). Subsequently, the crude biopolymer was purified by using the saturated barium hydroxide to minimize the impurities. Finally, the effect of different drying techniques on the flow characteristics and functional properties of the purified biopolymer was investigated. The present study elucidated the main functional characteristics such as flow characteristics, water- and oil-holding capacity, solubility, and foaming capacity.

**Results:**

In most cases except for oven drying, the bulk density decreased, thus increasing the porosity. This might be attributed to the increase in the inter-particle voids of smaller sized particles with larger contact surface areas per unit volume. The current study revealed that oven-dried gum and freeze-dried gum had the highest and lowest compressibility index, thus indicating the weakest and strongest flowability among all samples. In the present work, the freeze-dried gum showed the lowest angle of repose, bulk, tapped and true density. This indicates the highest porosity degree of freeze dried gum among dried seed gums. It also exhibited the highest solubility, and foaming capacity thus providing the most desirable functional properties and flow characteristics among all drying techniques.

**Conclusion:**

The present study revealed that freeze drying among all drying techniques provided the most desirable functional properties and flow characteristics for durian seed gum.

## Background

Natural carbohydrate biopolymers from plant sources provide a broad range of functional properties. They are appropriate alternatives to the synthetic biopolymers due to their biocompatibility, low toxicity, and low price as compared to synthetic biopolymers. Natural carbohydrate biopolymers are usually originated from nonpolluting renewable sources for the sustainable supply with a broad range of functional properties. They are mainly used for many applications as drug delivery carrier and binder, emulsifier, thickener, suspending agents and etc. The physicochemical and functional properties of natural plant-based biopolymers are extensively influenced by many factors such as the chemical composition and molecular structure of the biopolymer. On the other hand, the extraction, purification, drying and/or further modification processes can significantly affect the chemical composition and molecular structure, thereby influencing the functional properties of biopolymers.

Drying process is a critical food operation because it may induce undesirable changes in the texture, density and porosity, and sorption characteristics and overall quality of the dehydrated product [1]. Since, the removal of a large portion of moisture from food takes place during the drying process, therefore final characteristics of the dried product are extensively influenced by type and condition of the drying process [2]. The same raw material may end up as a completely different product, depending on the type and conditions of the drying process. The most common used drying techniques for various plant gums included oven drying [3], spray drying [4], freeze drying [5], and vacuum drying [6]. It should be noted that the drying process at high temperature for long time may result in the degradation of flavor compounds, color and nutrients of the dehydrated product, thus reducing the quality and overall acceptability of the final product [1,7].

The biopolymer from durian seed has a polysaccharide-protein structure. D-galactose and glucose were the most abundant monosaccharide in the carbohydrate profile of durian seed. The sugar analysis also revealed the presence of low content of arbinose and xylose in the chemical structure of durian seed gum [8]. As reported in previous studies [8,9], different extraction and further processing conditions significantly (p < 0.05) affected the chemical composition and molecular structure of the heteropolysaccharide-protein polymer from durian seed gum. This could be responsible for the considerable changes in emulsifying capacity, rheological and functional properties of the biopolymer from durian seed [10-13]. Previous study [11] revealed that the natural polymer from durian seed gum had the approprite interfacial activity (or emulsifying property) in oil in water (O/W) emulsion. This interfacial activity could be due to the presence of a low content of the proteineous constituent (< 4%) present in durian seed gum [8]. In addition, the heteropolysaccharide-protein polymer from durian fruit seed showed relatively low thickening properties in the aqueous solution [14]. This might be due to its relatively low molecular weight structure [9].

It is necessary to have sufficient information on flowability characteristics of the biopolymer in the powder and liquid form. Although, physicochemical and functional properties of durian seed gum have been extensively studied [10-14], but there is no similar study investigating the foaming properties and flowability characteristics (i.e. compressibility, bulk, tapped and true density) of durian seed gum as a function of drying conditions. The main goal was to investigate the effect of different drying techniques on chemical properties and flowability characteristics of durian seed gum. The current study helps the manufacturer for better understanding the functional characteristics of durian seed gum as a function of different drying conditions. It also provides helpful information regarding the most efficient drying technique for the preparation of durian seed gum. To the best of knowledge, the effects of different drying techniques on the chemical properties and flow characteristics of natural biopolymer from durian seed have not been reported on date. Different drying techniques (i.e. oven drying, spray drying, freeze drying and vacuum drying) were chosen based on the preliminary study and previous litrature [3-7,9]. The efficiency of different drying techniques was determined by assesing flow characteristics, water- and oil-holding capacity (WHC and OHC), solubility and foaming capacity of differently dried biopolymers.

## Results and discussion

### Bulk, tapped and true density

**Figure 1:**
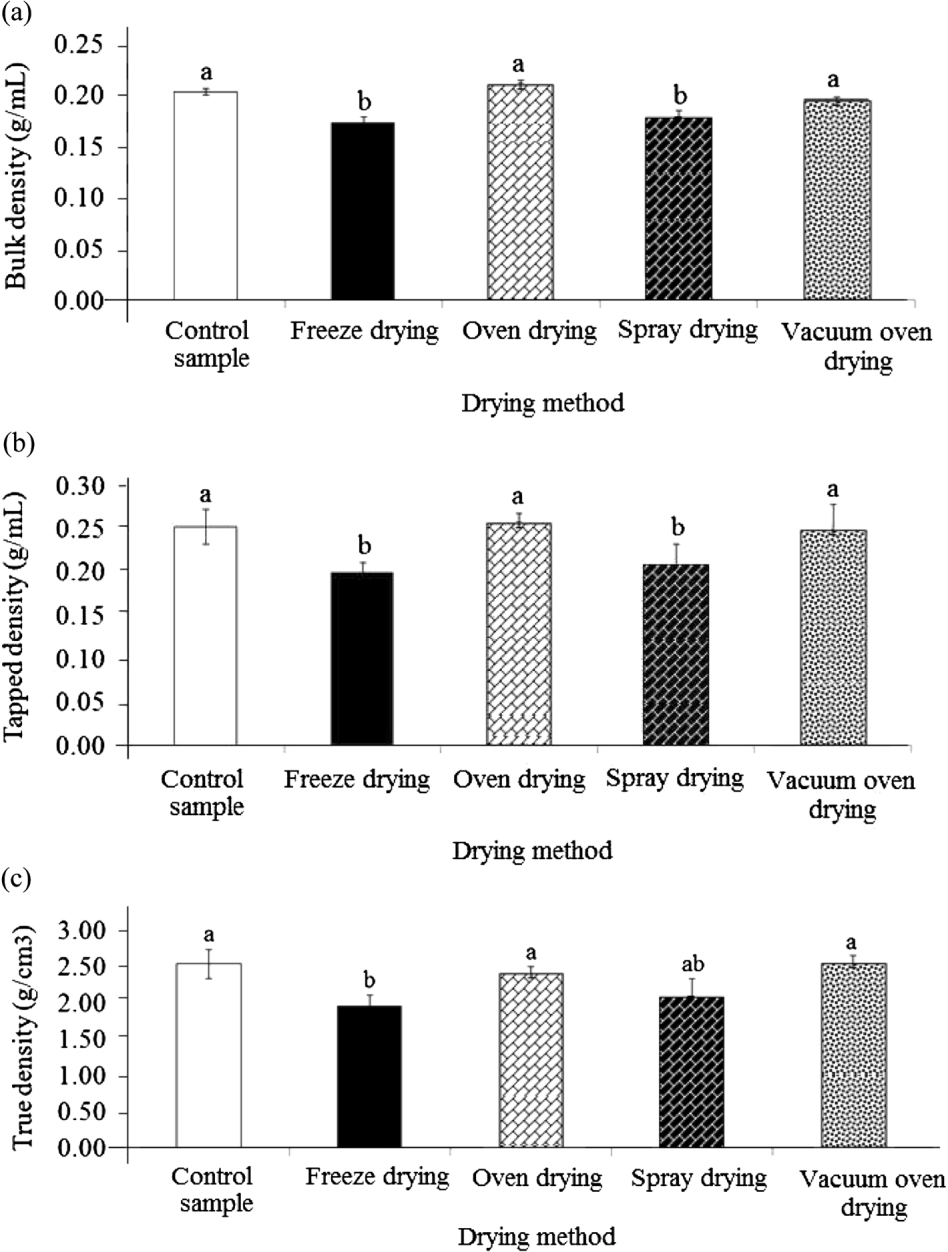
Effect of different drying methods on (a) the bulk density, (b) tapped density, and (c) true density of durian seed gum.

The physical properties such as bulk density, granule density, and inter-space porosity, wetting ability, particle size and distribution are critical parameters for controlling the quality of the powder. The density is a critical parameter affecting the functional properties of the powder. The bulk and tapped densities provide a perspective from the packing and arrangement of the particles and the compaction profile of a material [15]. The drying process significantly (p < 0.05) influenced the bulk density of the seed gum powder (Figure 1a). The bulk density depends on the attractive inter-particle forces, particle size and number of contact positions [16]. As also stated by Singh et al. [17], the bulk density of the powder is primarily dependent on particle size, particle size distribution and particle shape. This might be the reason for the significant changes in the bulk density of durian seed gum. The bulk density of durian seed ranged from 0.173-0.203 g/mL, depending on the drying technique (Figure 1a). Previous researchers reported different bulk densities for various plant gums (Table 1). The freeze drying and spray drying significantly (p < 0.05) decreased the bulk density. In the present work, the oven-dried seed gum had the highest bulk density (Figure 1a). Among all drying techniques, the freeze-drying exhibited the highest reduction of the bulk density (Figure 1a). The bulk density is reversely associated with porosity ([1- (bulk density/granule density)] × 100) [18]. In fact, the substance with lower bulk density has the higher porosity and vice versa.

**Table 1:** Physicochemical properties of different plant gums

**Plant gum**	**Bulk density (g/mL)**	**Tapped density (g/mL)**	**True density (g/cm**^**3**^**)**	**Compressibility index (%)**	**Angle of repose (°)**
Air- dried Grewia gum^a^	0.160±0.00	0.200±0.01	2.0±0.01	20.20±1.20	30.40±0.50
Freeze- dried Grewia gum^a^	0.140±0.00	0.170±0.01	1.7±0.01	21.40±0.84	32.60±1.03
Guar gum^b^	0.474±0.06	0.546±0.05	-	13.05±0.7	19.24±0.05
Dioclea gum^b^	0.564±0.05	0.706±0.01	-	20.14±0.2	25.36±0.1
Reflexa gum^b^	0.174±0.06	0.225±0.05	-	22.75±0.5	27.30±0.06
Afzelia Africana^c^	0.610 ± 0.05	0.710 ± 0.00	1.7	14.08	10.61 ± 1.17
Tragacanth^c^	0.640 ± 0.00	0.740 ± 0.01	-	13.51	21.77 ± 2.74
Gum Arabic^d^	0.61	0.86		28.42	-
Mangifera gum^d^	0.74	0.92	-	19.56	26.75

^a^ Nep and Conway [19]; ^b^ Builders et al. [20]; ^c^ Martins et al. [21]; ^d^ Kumar Singh et al. [15].

In the current study, the freeze-dried gum followed by the spray-dried gum had the lowest bulk density, thus providing the highest porosity among all dried gums. The significant reduction in the bulk density might significantly affect the solubility of the freeze-dried and spray dried gums. The bulk density of different dried seed gums was comparable with that of reported for grewia gum (0.140-0.160 g/mL) [19], and reflexa gum (0.174±0.06 g/mL) [20]. Conversely, it was lower than the bulk density reported for guar gum (0.474±0.06 g/mL), dioclea gum (0.564±0.05 g/mL) [20], afzelia Africana (0.610±0.05 g/mL), tragacanth (0.640±0.00 g/mL) [21], gum Arabic (0.61), and mangifera gum (0.74 g/mL) [22] (Table 1). The total volume of inter-particle voids can change with drying and packing processes; therefore, tap density should be measured to rectify this matter. The tap density is one of main characteristics of a powder which is the maximum packing of a powder achieved under the influence of well defined, externally applied forces. It indicates the volume of a mass of sample after inducing a closer packing of particles by tapping the container. Goldfarb and Ramachandruni [23] illustrated that the tapped density should be measured for two crucial reasons. Firstly, the tapped density value is more reproducible than the bulk value. Secondly, the flow characteristic of a powder is inferred from the ratio of these two measured densities. In the current study, the tapped density ranged from 0.199 to 0.258 g/mL, depending on the drying technique (Figure 1b).

As reported by previous researchers, various plant gums showed different flowability characteristics (Table 1). The results exhibited the significant (p < 0.05) effect of freeze and spray drying methods on the tapped density of durian seed gum (Figure 1b). This study revealed that the freeze-dried seed gum had the least tapped density. On the other hand, the oven-dried seed gum followed by the control sample contained the highest tapped density, thus indicating the least porosity among all dried samples (Figure 1b). The tapped density results showed that the freeze dried and spray dried seed gums are more porous than the other samples. The true density can be equal to the theoretical density of the material, depending on the molecular arrangement of the material. In fact, the true density indicates whether the material is close to a crystalline state or the proportions of a binary mixture. The results indicated that the drying process significantly (p < 0.05) affected the true density of the gum (Figure 1c). The significant changes in the tapped and true density of the dehydrated products significantly influence their overall quality [24]. In the current study, the true density varied from 1.98 to 2.50 g/cm^3^ (Figure 1c). This value was comparable with the true density reported for grewia gum (1.7-2.0 g/cm^3^), and afzelia Africana gum (0.17 g/cm^3^) [21] (Table 1). As shown in Figure 1c, the spray drying and freeze drying led to reduce the true density. This observation was in agreement with that of reported by Nep and Conway [19] for grewia gum (Table 1). They also observed that the true density of air–dried grewia gum was higher than the true density of the freeze-dried grewia gum (Table 1). In this study, the freeze drying caused the highest significant (p < 0.05) reduction in the density of durian seed gum (Figure 1c).

### Compressibility index and angle of repose

**Figure 2:**
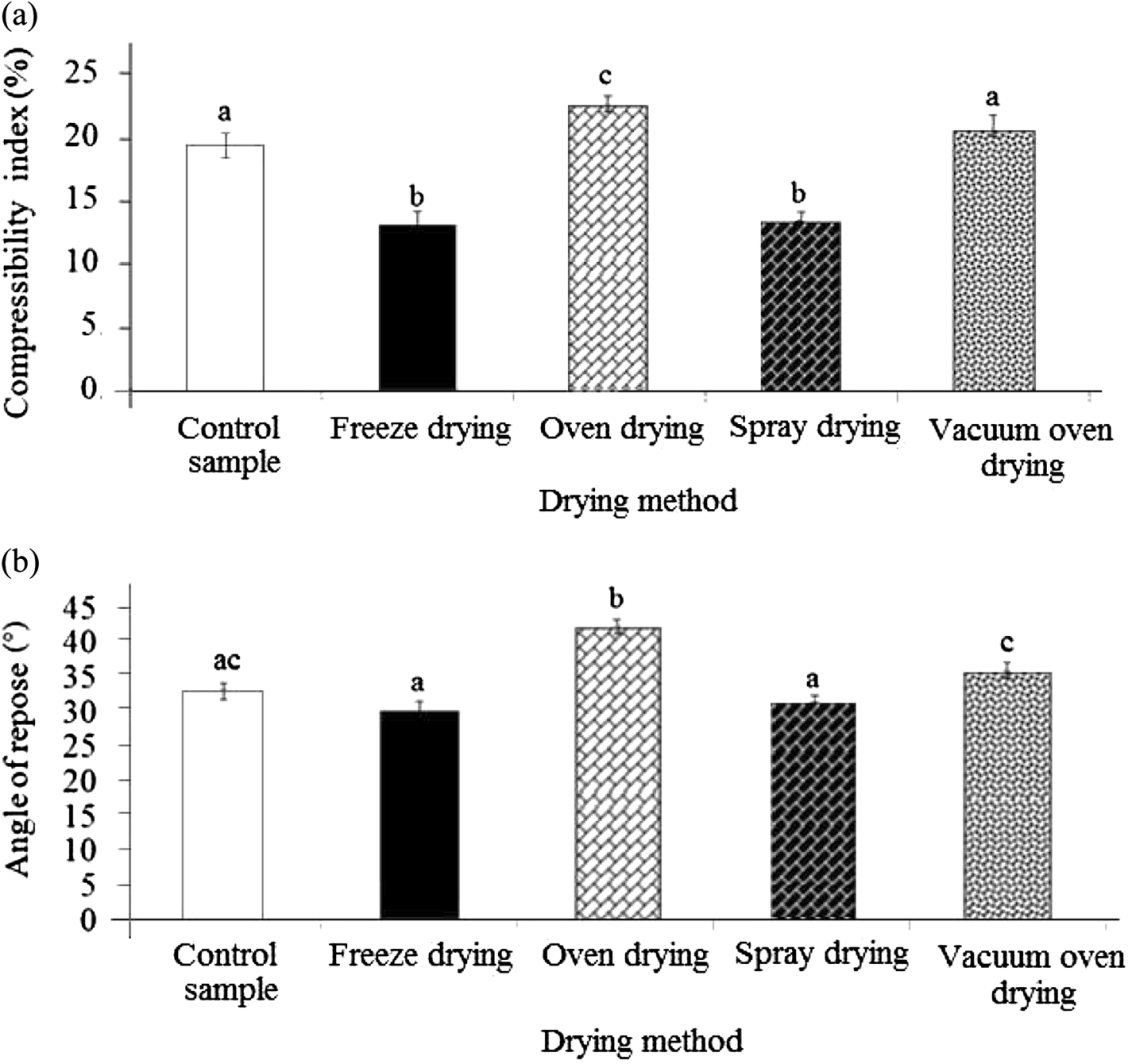
Effect of different drying methods on (a) the compressibility index, and (b) angle of repose of durian seed gum.

The effects of different drying techniques on the compressibility index and angle of repose of durian seed gum were shown in Figure 2a, b. In the present study, the compressibility index of durian seed ranged from 13.06 to 22.85%, depending on the drying technique (Figure 2a). As stated by previous researcher [25], the excellent or poor flowability characteristics of the powder can be assessed by determining its compressibility index (Table 2). If the compressibility index is less than 10%, this shows excellent flow. The low compressibility index (11–15%) indicates good flowability characteristics; while the relatively high compressibility index (16-20%) and very high compressibility index (> 31%) indicate fair and very poor flowability characteristics (Table 2) [25]. This indicated that the different dried-seed gums showed different flow characteristics from fair to good (13.06 to 22.85%). As recommended by Phani Kumar et al. [22], if the compressibility index varies from 15 to 25%, the modification of particle size distribution is advisable to reach the optimum performance and very good flow properties.

**Table 2:** **Flow properties based on compressibility index and angle of repose **[25]

**Compressibility index (%)**	**Angle of repose (°)**	**Flow character**
≤10	25-30	Excellent
11-15	31-35	Good
16-20	36-40	Fair
21-25	41-45	Passable
26-31	46-55	Poor
32-37	56-65	Very poor
>38	>66	Very, very poor

**Figure 3:**
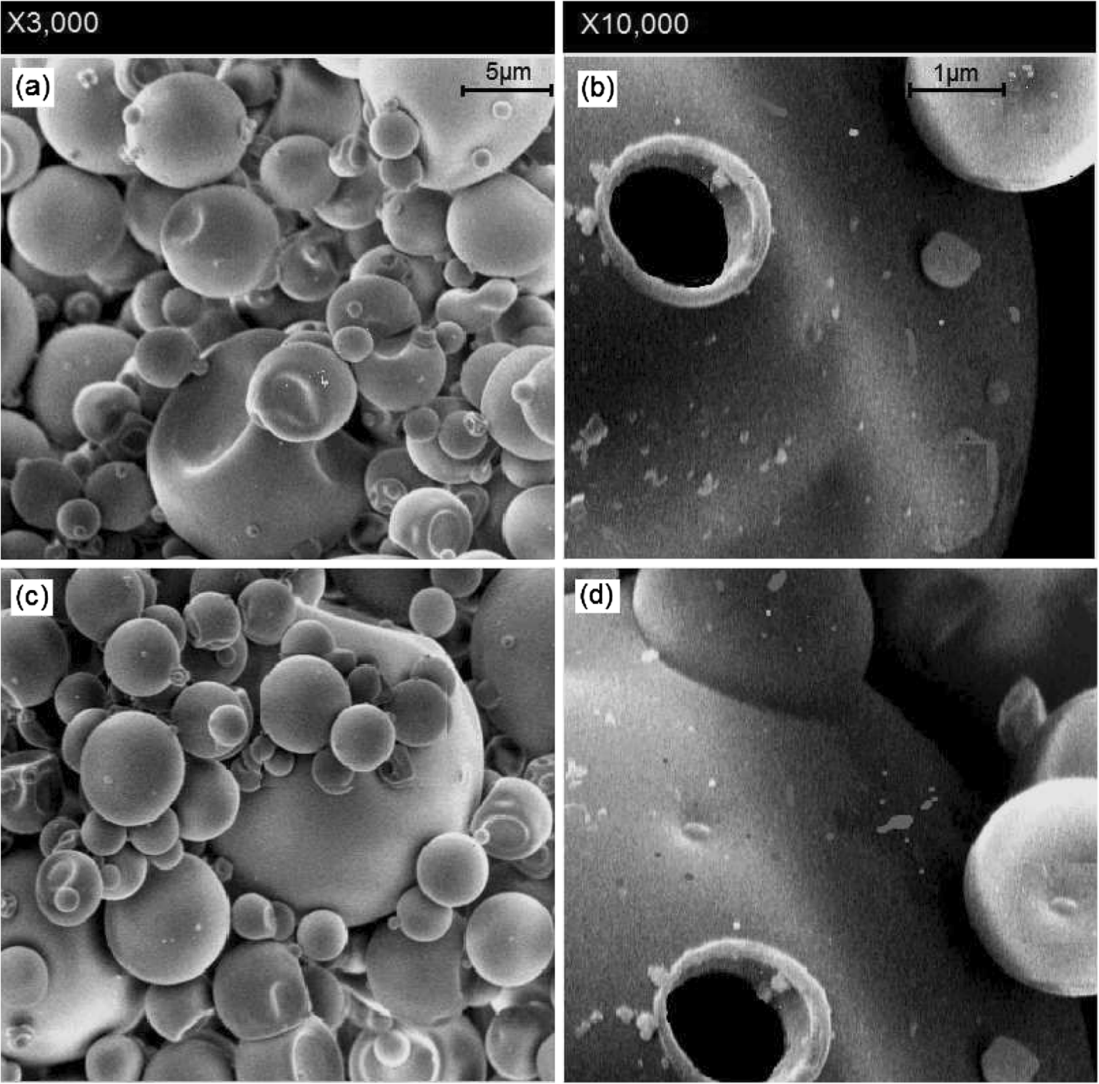
Scanning Electron Microscopy (SEM) images of the freeze-dried seed gum from general particle population (a, c) (X3,000) and a closer look (b, d) (X10,000).

Among all samples, the freeze-dried seed gum exhibited the lowest compressibility index (13.1%), thus indicating good flow characteristics (Figure 2a). Figure 3 displays SEM images of the freeze-dried gum showing the smooth skin-forming behavior of dried particles. The left side image shows some agglomerations which have been taken place during the drying process. Collisions are apparent between semi-dried particles (Figure 3). Collision between particles was expected to take place whilst some of the particles had been still liquid. Some of the larger particles show some “deflating”, due to softness of the shell during drying. The right side image is a close image showing a shell-type structure. This large magnification shows a large particle with a rough, porous surface, which is revealed under the smooth outer skin. The presence of porous particles is expected after the sublimation of ice crystals during freeze drying process.

As shown in Table 1, various plant gums exhibited different compressibility index and angle of repose, thus indicating different flow characteristics. The freeze-dried durian seed gum showed the least compressibility index, thus providing the most appropriate flowability characteristics among all dried gums. Conversely, the oven-dried durian seed gum had the highest compressibility index, thus indicating the weakest flowability among all samples (Figure 2a). The flow rate of a material depends upon many factors related to the particle structure and processing conditions. As stated by previous researchers [25], the compressibility and compatibility of a powder can affect its flow properties in the micro-scale through the adhesion forces between the particles. The angle of repose is also one of the critical features indicating the degree of flow characteristics of powder granules. The increase in angle of repose is associated with decreasing the flowability characteristics (Table 2). It is a measure of powder resistance to the flow under gravity due to frictional forces resulting from the surface properties of the granules [26]. As shown in Figure 2b, the different dried durian seed gums showed different levels of angle of repose ranging from 30.83-42.22°. This range was comparable with the compressibility index reported for grewia gum (30.40-32.60°). However, this value was higher than the angle of repose reported for dioclea gum (25.36±0.1°), reflexa gum (27.30±0.06°) [20], afzelia Africana (10.61 ± 1.17°), and tragacanth (21.77 ± 2.74°) [21] (Table 2).

As reported by Onunkwo [26], if the angle of repose decreased, the binding level of the granules increased. This might be due to the reduction in the cohesive forces of the larger granules formed at higher binding level [27]. The drying process had a significant (p < 0.05) effect on the angle of repose of durian seed gum, thus affecting its flow characteristics (Figure 2b). The significant effect of the drying process on the angle of repose was also reported by Nep and Conway [19]. The oven-dried gum exhibited the highest angle of repose; while the spray-dried gum and freeze-dried gum showed the lowest angle of repose among all dried samples (Figure 2b). Conversely, the oven drying resulted in the highest angle of repose (42.22°). Although, the oven drying is a low cost drying technique, but it results in passable to relatively poor flowability characteristics for durian seed gum. Nep and Conway [19] reported that the freeze drying resulted in higher angle of repose (or better flowability) than air drying for grewia gum.

### Solubility

**Figure 4:**
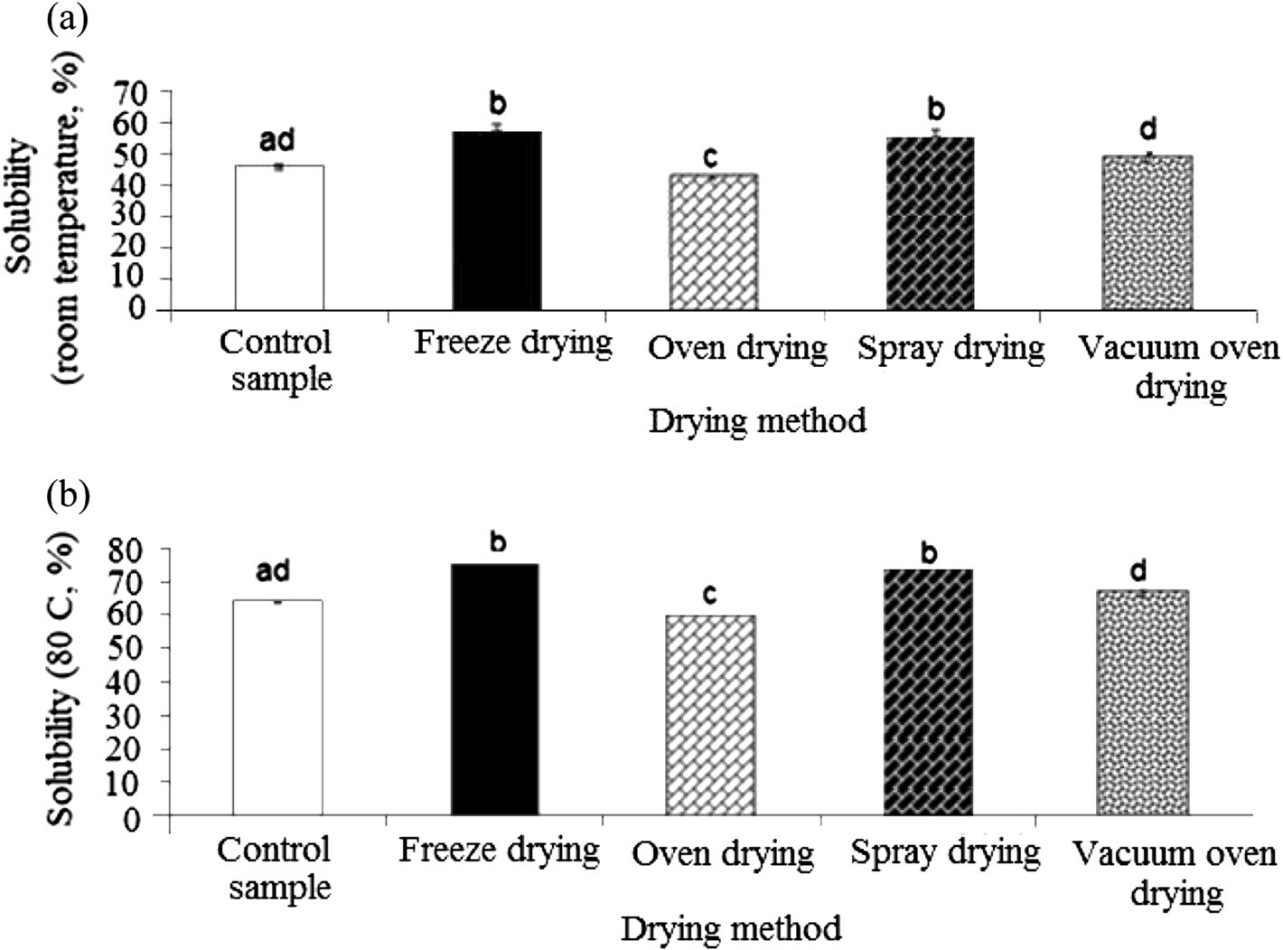
Effect of different drying methods on the solubility of durian seed gum at (a) the room temperature, and (b) elevated temperature (80°C).

Solubility is the most reliable criterion to evaluate the behavior of powder in aqueous solution. This parameter is attained after the powder undergoes dissolution steps of sinkability, dispersability and wettability. The present study showed that the drying process significantly (p < 0.05) influenced the solubility of durian seed gum (Figure 4a, b). This could be explained by the significant (p < 0.05) effect of the drying process on the chemical composition of durian seed gum as reported in the previous study [9]. Previous study [9] revealed that drying process significantly (p < 0.05) influenced the content of galactose, glucose, arabinose and xylose present in the chemical structur of durian seed gum. This might be responsible for the significant (p < 0.05) changes of solubility. On the other hand, drying process significantly (p < 0.05) influenced the protein content and amino acid composition of durian seed gum as reported previously [9]. This might be another reason for the significant (p < 0.05) changes of solubility as a function of drying conditions. As reported earlier [9], drying process significantly affected the content of leucine, lysine, aspartic acid, glycine, alanine, glutamic acid, valine, proline, serine and threonine present in the chemical structure of durian seed gum. In addition to the chemical composition, drying process had a considerable effect on the molecular structure of durian seed gum as reported previously [9]. This might be also responsible for significant (p < 0.05) changes of the solubility of the durian seed gum as a function of different drying conditions. Durian seed gums exhibited different solubility levels at the room temperature (43.0-57.5%) as compared to the control sample (46%), depending on the drying method (Figure 4a). The solubility of different-dried durian seed gums the room temperature was similar to that of reported for carob gum (~50%, at 25°C) [28]. Nep and Conway [19] also found that the solubility of grewia gum was significantly (p < 0.05) influenced by the drying process. They reported different degree of solubility (0.1-0.3 mg/mL) for grewia gum. This could be explained by the fact that different drying techniques resulted in different molecular weights, thus varying the solubility [19].

In the current study, the spray-dried and freeze-dried gums showed remarkably higher solubility than the control sample, oven-dried (105°C) and vacuum oven-dried gums (Figure 4a). This could be also due to the significant size reduction, more particle size uniformity and complete conversion of particle to more soluble amorphous form. Corrigan et al. [29] also reported that high energy amorphous form caused by spray drying led to improve the functional properties of powder such as the enhanced solubility and faster dissolution rate. The high solubility of freeze-dried and spray dried gums could be due to their low bulk density (or high porosity). The difference could be also explained by the significant effect of the drying process on the monosaccharide composition present in the backbone and side chains of the gum molecular structure. As explained by Kuntz [30], the physicochemical properties of gum (such as WHC, viscosity, hydration and solubility) are attributed to its molecular structure (i.e. the type and number of monosaccharides, type, number and location of the linked glycosidic group).

The current study revealed that purified durian seed gum also showed a similar trend of solubility at room and high temperature (80°C). The presence of the protein fraction along with the monosaccharide structure of gum most probably affects its water solubility [31]. The structure of protein significantly affects it reaction with water molecules. It is hypothesized that the drying process significantly affects the position of hydrophobic and hydrophilic amino acids in the interior or exterior layer of the protein molecule, thus affecting the solubility of the protein fraction [32]. The different sets of strong covalent bonds or weak non-covalent bonds (i.e. such as Van der Waals attractions, hydrogen and ionic bonds) that form between one part of the protein chain and another can significantly alter the solubility of the protein. The interaction between water and protein molecules can build up new hydrogen bonds with the amide nitrogen and carbonyl oxygen of peptide bonds. These interactions result in the further weakness nearby hydrogen bonds, thus affecting the solubility and functional properties of protein fraction [32]. The resulted indicated that the solubility of durian seed gum was relatively high at the elevated temperature (80°C) (Figure 4b). Dakia et al. [28] also reported that the solubility of carob gum reached the maximum level (~70–85%) at 80°C as compared to the low solubility (~50%) at 25°C. The solubility of durian seed gums at the elevated temperature (80°C) was significantly (p < 0.05) different its solubility at the room temperature. It ranged between 60 to 75.2% as compared to the control sample (63.7%). The high solubility of freeze-dried gum may be attributed to the high content of hydrophilic fraction and soluble materials as well as its low cross-linking [33]. The high solubility of the freeze-dried gum might be also interpreted by the fact that the freeze drying could result in the low bulk density and high porosity.

### Water- and oil-holding capacity (WHC and OHC)

The results indicated that the drying process significantly (p < 0.05) influenced the capacity of water absorption (WHC) of durian seed gum. This could be due to the significant effect of the drying process on the chemical composition and molecular structure of durian seed gum [9]. Mishra et al. [34] also reported the significant differences between the chemical composition of polysaccharide gums as a function of the drying process. Many hydrocolloids have side units such as sugar units, carboxyl groups, sulfate groups or methyl ether group which influence the functional properties of the hydrocolloid. Water molecules are oriented around hydroxyl groups of sugar units and around anionic groups presenting on some gums. They move around with the gum molecules, leading to swelling and increasing the volume [35]. The peripheral polar groups and central hydrophobic stem of polysaccharide molecules give different interactions with water and electrolytes depending on their compositions [36].

**Figure 5:**
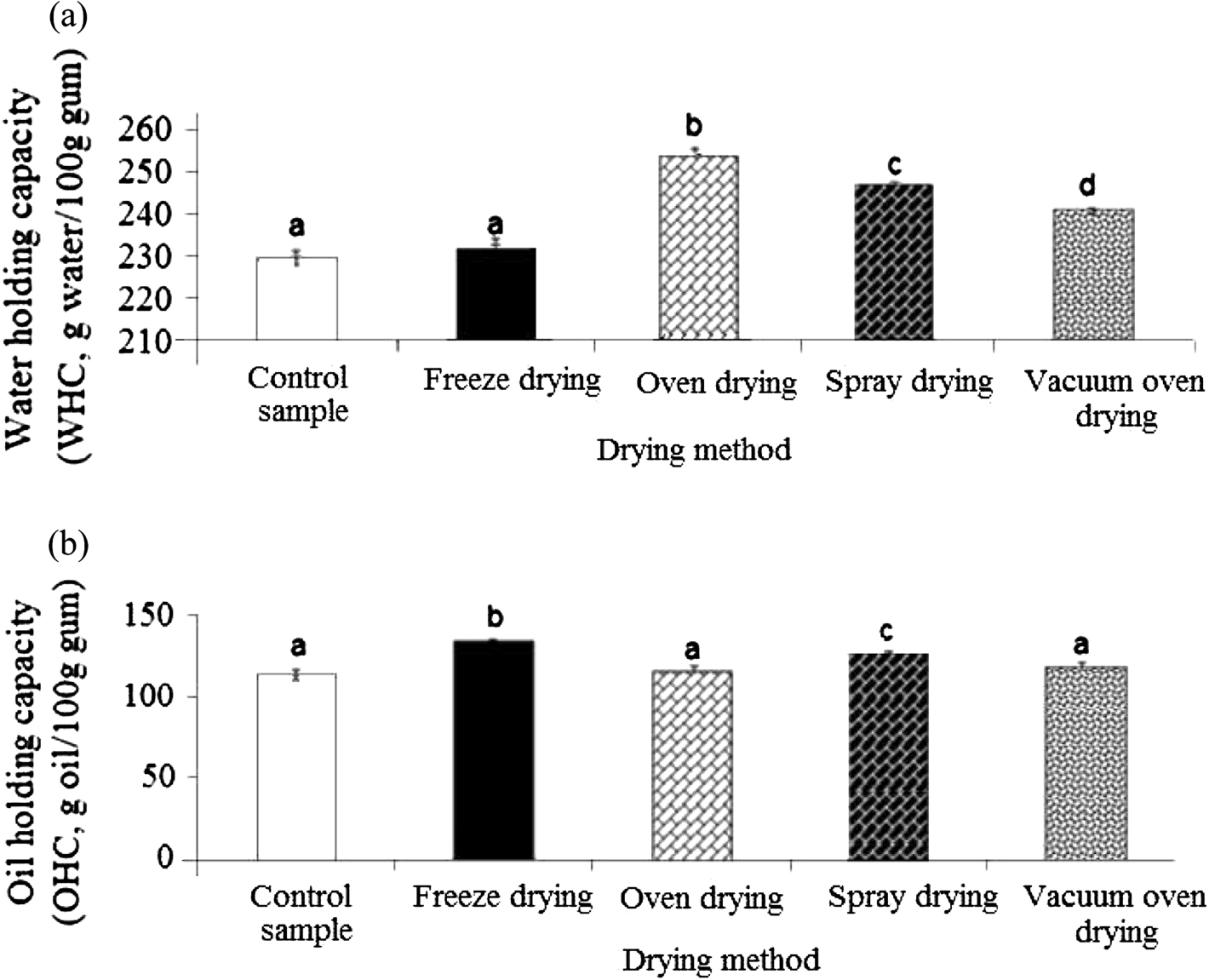
Effect of different drying methods on (a) water-holding capacity (WHC) and (b) oil-holding capacity (OHC) of durian seed gum.

In the current study, WHC of different-dried durian seed gums varied from 232.8-254.8 (g water/100 g gum) as compared to the control sample (229.6 g water/100 g gum) (Figure 5a). This value was comparable with WHC reported for fibre-rich fractions (FRFs) (237–320 mL/100 g), but lower than the WHC of cellulose (381 mL/100 g) from the defatted passion fruit seed [37]. Torio et al. [31] reported a relatively low water-holding capacity (42.55-47.28%) for galactomannan from sugar palm (*Arenga saccharifera *Labill.) endosperm. The oven-dried gum showed the highest significant (p < 0.05) capacity of water absorption (254.8 g water/100 g fibre) among all samples. This value was comparable with WHC reported for citrus husk DF (360 g water/100 g fibre) and pineapple peel dietary fiber (DF) (350 g water/100 g fibre) [38]. However, it was much lower than WHC reported by Adams et al. [39] for some agricultural by-product from wheat bran (660 g water/100 g fibre), apple wastes (1170 g water/100 g fibre), and orange wastes (1620 g water/100 g fibre).

The spray-dried gum also exhibited a significant (p < 0.05) higher WHC than the control, freeze-dried and vacuum oven-dried gums; while the control sample provided the lowest significant (p < 0.05) WHC among all samples (Figure 5a). It was found that the freeze-drying did not significantly (p > 0.05) influence the capacity of water absorption of durian seed gum. It should be noted that WHC of gum does not only depend on the functional group of polysaccharide fraction that are hydrophilic groups, but also on the protein fraction present in the gums. They also contain specific functional groups that are able to bind water molecules [40]. The capacity of water absorption also depends on the number and nature of the water-binding sites [37]. Chou and Morr [41] also demonstrated that WHC varies as a function of several factors such as the hydrophilic–hydrophobic balance of amino acids in the protein molecule, lipid and carbohydrate fractions associated with the protein. The results showed that the drying process significantly (p < 0.05) influenced the oil-holding capacity (OHC) of durian seed gum (Figure 5b). The different-dried durian seed gums exhibited different capacities of oil absorption. This could be interpreted by the significant effect of the drying process on the hydrophobic fraction (i.e. lipid and protein fractions) present in the structure of durian seed gum. As also stated by Hayta et al. [42], the oil absorption capacity of food material depends on the type and content of hydrophobic fraction present in the matrix structure. The presence of trace fatty acid and hydrophobic amino acid in the structure of durian seed gum may be responsible for its tendency for oil absorption. The existence of several non polar side chains may bind the hydrocarbon chains of oil, thereby resulting in higher OHC [43].

As shown in Figure 5b, OHC of different-dried durian seed gums varied within the range of 114.9 to 132.8 (g oil/100 g gum) as compared to the control sample (113.2 g oil/100 g gum). This value was comparable with OHC reported for orange byproduct fibres (90–130 g/100 g) [44], but lower than that of reported for fibre-rich fractions (FRFs) (207–372 g/100 g) from the defatted passion fruit seed [37]. The current study revealed that the drying led to increase the capacity of oil absorption as compared to the control sample. As shown in Figure 5b, the freeze-dried gum gave the highest significant (p < 0.05) OHC among all samples. This might be due to the lower destructive effect of the freeze drying on the hydrophobic fraction present in durian seed gum than the effects induced by other drying techniques. The oven-dried (105°C) gum had the lowest capacity of oil absorption. This could be due to the thermal oxidation (105°C) of trace lipid fraction present in the gum structure.

### Foaming capacity

**Figure 6:**
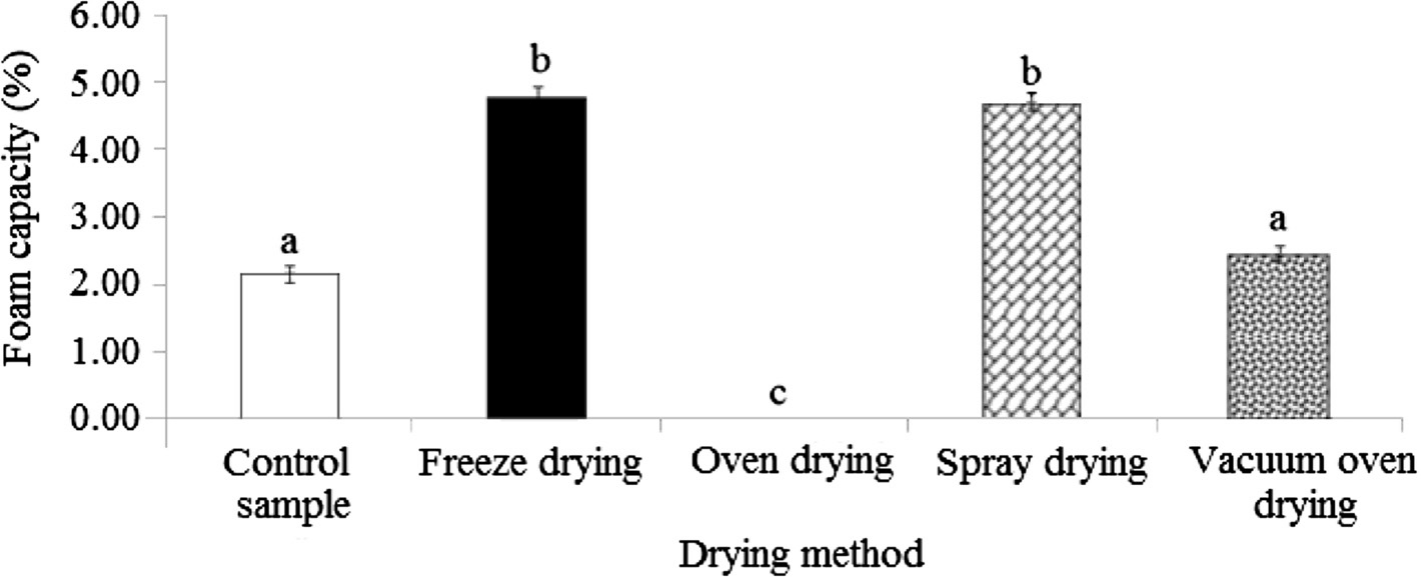
Effect of different drying methods on the foaming capacity of durian seed gum.

The present study showed that the drying process significantly (p < 0.05) influenced the foaming capacity of durian seed gum (Figure 6). As shown in Figure 6, different dried durian seed gums exhibited different levels of foam capacity (0.00-4.78%), depending on the drying method (Figure 6). Our preliminary study revealed the presence of the protein fraction (< 4%) in the molecular structure of durian seed gum [9]. It was hypothesized that the foaming properties of the natural biopolymer from durian seed could be due to the presence of the protein fraction along with its carbohydrate structure. In protein/polysaccharide systems, the possible phase separation affects the foam stability. When air is injected into a solution containing the protein-polysaccharide biopolymer, the entrapment in the foam of bubbles occurs as a result of the absorption of protein molecules at the bubble surface. The basic requirements for the proper foaming properties of the protein fraction are its ability to: (a) adsorb rapidly at the air–water interface during bubbling; (b) undergo rapid conformational change and rearrangement at the interface, and (c) form a cohesive viscoelastic film via intermolecular interactions [45]. The rapidly adsorption of the protein at the air–water interface and conformational rearrangement at the interface are associated with the appropriate foaming ability.

The freeze-dried gum and oven-dried gum showed the highest and lowest foaming capacity among all samples. It was hypothesized that the freeze drying might result in the least destructive effect on the protein structure, thus preserving the functional properties induced by the protein fraction present in the molecular structure of durian seed gum. On the other hand, the poor foaming capacity of oven-dried seed gum might be due to the thermal denaturation of the protein fraction. In fact, different drying techniques resulted in different protein content, and molecular weight [9]. In the current study, the freeze-dried and the spray-dried gums showed remarkably higher foaming capacity than the other samples (Figure 6). In the current study, the foam caused by stirring the gum solution was not stable for the long time. This could be due to the liquid drainage from the foam. In fact, the air bubbles are spherical, and lamellas containing large amounts of water are thick, when the foam is initially formed. The lamella becomes thin by the time, and the liquid drainage from the foam starts. Subsequently, air bubbles pack closer, and assume polyhedral shapes. In fact, the liquid drainage from the thin lamellae is the main destabilizing force allowing the bubbles to become closer. Consequently, the large bubbles are growing at the disbursement of small ones, when the film membrane becomes permeable; the disproportionation reaction occurs [46]. It should be noted that disproportionation is a typically a redox chemical reaction, where a single element is simultaneously oxidized and reduced. Finally, the film membrane at the air–foam interface ruptures, leading to collapse the foam [46].

## Conclusions

The main objective of the current study was to investigate the effect of different drying techniques on flowability characteristics and functional properties of the natural carbohydrate polymer from durian fruit seed. The present work revealed that the freeze drying provided the most suitable flowability characteristics for durian seed gum. The freeze dried gum showed the highest porosity, solubility and foaming capacity among differently dried seed gums. This might be due to the least thermal degradation, which probably resulted in less compact structure than other samples. On the other hand, the spray drying also also produced durian seed gum with the appropriate flow characteristics and functional properties as compared to freeze drying. Since, freeze dryer is an expensive method to apply in the commercial scale; spray dryer may be a proper alternative technique for producing durian seed gum. Although, the oven drying is a low cost drying technique as compared to spray drying and freeze drying, but the current study reveals that it provides a low quality durian seed gum with undesirable functional properties (i.e. poor flowability characteristics, low foaming capacity, and relatively low solubility and oil holding capacity). It was hypothesized that the oven drying at the elevated temperature (105°C) might have caused the collapse in the gum structure. The thermal degradation possibly induced by high drying temperature might result in more compact and rigid powder with the low porosity. The present work suggests a further study to produce durian seed gum with mono-dispersed particles and explore its dissolution mechanism.

### Experimental

#### Chemicals and materials

Isopropanol, ethanol (95% and 99.9%), acetone, hydrochloric acid, saturated barium hydroxide, sodium hydroxide, acetic acid were purchased from Fisher Scientific (Pittsburgh, PA, USA). Durian (*D. zibethinus*) fruit was purchased from the local market (Selongor, Malaysia). Ripened durian fruits were selected based on the size uniformity and free of visual defects. The fruits were then de-husked (cut open the rind), by cutting along the suture on the back of the lobules. Durian seeds were removed, cleaned and rinsed thoroughly with sterile distilled water. The seed was partially dried by the air circulation at the ambient temperature for one overnight. The dried seeds were then packed in plastic bags and stored in a dry and cool place (10 ± 2°C) until the extraction process [47]. All the experiments were performed with deionized water.

### Chemical extraction process

Chemical extraction was performed according to the method described by Singh et al. [17] with the minor modification. Durian seed were washed and chopped into small pieces. Then, it was air dried by using the air circulation before milling into flour. The cold extraction was used to extract the oil from durian seed flour in order to avoid the thermal degradation. The defatting process was carried out successively using hexane and isopropanol (60:40) at the room temperature (25 ± 1°C). Preliminary trials showed that the solvent mixture containing hexane and isopropanol (60:40) was the most efficient solvent for defatting process among all studied solvents (i.e. petroleum ether, hexane, isopropanol and ethanol). The solvent residue was removed by centrifugation at ~3000 rpm for 15 min using the Beckman Coulter Centrifuge (JA-14, Beckman Coulter GmbH, Krefeld, Germany). Then, defatted-durian seed flour (1 kg) was exhaustively decolored using ethanol at the decoloring time 120 min. The decolorized seed flour was vacuum filtered and then soaked in 1% aqueous acetic acid for 1.5 h at the ambient temperature. Then, the slurry was filtered by using Nylon cloth filter and the filtrate was precipitated with 95% ethanol. The precipitated slurry was washed three times using absolute ethanol (99.9%) to achieve very light brown amorphous crude gum [48]. The crude gum was collected and oven dried at 40°C.

### Purification process

The crude seed gum was purified through barium complexing according to the method described by previous researchers [10,49]. In this method, the gum solution (2.5% w/v) was prepared by dissolving 2.5 g of the crude durian seed gum in 100 mL of water and stirring for 12 h at 60°C. Then, the gum solution was precipitated with saturated barium hydroxide solution. The precipitate was separated by the Beckman centrifuge at 3500 rpm for 15 min. Then, the precipitate was stirred with 1 M acetic acid for 8 h and again centrifuged. The supernatant was precipitated with 90% ethanol. The precipitate was washed with 95% ethanol and oven dried at 40°C.

### Drying process

#### Oven drying

Purified seed gum was dried according to the method described by the previous researchers [3] with minor modification. The seed gum solution (10% w/v) was prepared by dissolving 10 g of the purified durian seed gum 100 mL of deionized water. The coarse gum solution was homogenized using a high pressure homogenizer (APV, Crawley, UK) for two cycles at different pressure levels (30 and 25 MPa). Then, the homogenized-gum solution (10% w/v) was dried by using the oven dryer at 105°C for 3 h. The dried sample was dry milled and passed through a 1.0 mm sieve [10]. Then, the milled powder was weighed and stored in the airtight container before further analysis.

#### Vacuum oven drying

The vacuum oven drying was employed according to the procedure described by Wang et al. [3] with some modification. The coarse gum solution (10% w/v) was prepared and then homogenized using the same homogenization process (at 30 and 25 MPa). Then, the homogenized-gum solution was dried by using a vacuum-dryer at 60°C for 24 h. The vacuum and temperature were maintained at 5 Psi and 60°C, respectively. The dried sample was then milled and passed through a 1.0 mm sieve [9,10]. Finally, the milled powder was packed in the airtight container prior to the analysis.

#### Spray drying

Spray-dried seed gum was produced according to the method described by the previous researchers [19] with minor modification. Initially, the coarse gum solution (10%) was homogenized using the same processing condition as described earlier. The homogenized-gum solution (10%) was spray-dried by using a co-current spray dryer (Niro model 2000A, Niro Atomizer, Copenhagen, Denmark) equipped with a vanes centrifugal atomizer. The spray drying was performed at the pressure, inlet and outlet temperatures of 552 Kpa, 160°C and 80–85°C, respectively. The flow rate was controlled by adjusting the feed rate (50 mL/min) through the atomizer with a peri- staltic pump. Finally, the spray-dried gum was collected at the bottom of the cyclone [10]. The spray-dried seed gum was milled and passed through a 1.0 mm sieve and packed in the airtight containers before further analysis.

#### Freeze drying

The freeze-dried seed gum was produced according to the procedure described by Nep and Conway [19] with some modification. Initially, the coarse gum solution (10%) was homogenized using the same homogenization condition as described earlier. Then, the homogenized-gum solution (10%) was placed in Petri dishes and pre-frozen at −20°C for 24 h prior to freeze-drying process. The freeze drying was carried out by using a freeze dryer (Labconco Freezone 18, Model 77550, MO, USA). The Petri dishes were then transferred into freeze drier chamber and frozen at −40°C for 48 h [9]. The freeze-dried gum was dry milled and passed through a 1.0 mm sieve and packed in the airtight containers prior to the analysis.

### Analytical test

#### Bulk, tapped and true density

The bulk and tapped density of durian seed gum was investigated according to the method described by previous researchers [19]. Durian seed gum powder (10 g) was weighed into a 100 mL measuring cylinder and, the volume was recorded as the bulk volume (V). The bottom of the cylinder was raised about 10 cm above the slab and made to fall on the platform continuously for 100 taps, and the volume was recorded as tapped volume (V_T_) [50]:

(1) $$\text{Bulk}\;\text{density}\;\left(D_{B}\right)=\frac{\text{Mass}}{V}$$

(2) $$\text{Tapped}\;\text{density}\;\left(D_{T}\right)=\frac{\text{Mass}}{V_{T}}$$

True density was determined by the liquid displacement method at 25°C according to the method described by previous researchers [22]. True density was calculated by dividing the weight of the insoluble solid material by the weight of the liquid it displaces. The clean, dry 50 mL density bottle was weighted (W_1_), and then filled with water. Then, the top of the bottle was dried with filter paper, and weighed as W_2_. The procedure was repeated using benzene to obtain the weight (W_3_) of the bottle containing benzene. In this experiment, benzene was used as the displacement liquid. About 3 g of the dried seed gum was transferred to the density bottle and weighed as (W_4_). Then, the density bottle was filled with benzene and weighted (W_5_). True density was calculated using the following equations [22]:

(3) $$\text{Density}\;\text{of}\;\text{benzene}=\frac{\left(W_{3}-W_{1}\right)\times 0.9971}{\left(W_{2}-W_{1}\right)}$$

$$\text{Density}\;\text{of}\;\text{water}\;\left(25\mathrm{{}^{\circ}C}\right)=0.9971\;g/\text{cc}$$

(4) $$\begin{array}{l} \text{True}\;\text{density}\;\text{of}\;\text{the}\;\text{gum}\\ \;=\frac{\left(W_{4}-W_{1}\right)}{\left[\frac{\left(W_{3}-W_{1}\right)}{\rho }\right]-\left[\frac{\left(W_{5}-W_{4}\right)}{\rho }\right]} \end{array}$$

True density measurement is also one of the most economical simple methods for determining the powder quality. It is the density of the solid material, which is calculated by the mass of a particle divided by its volume, excluding open pores and closed pores [23].

#### Compressibility index

Compressibility index of durian seed gum was examined based on the compressibility index [51]:

(5) $$\begin{array}{l} \text{Compressibility}\;\text{index}\;\left(\%\right)\\ \;=\frac{\text{Tapped}\;\text{density}-\text{Bulk}\;\text{density}}{\text{Tapped}\;\text{density}}\times 100 \end{array}$$

#### Angle of repose

The angle of repose (θ) was determined by using the method described by Nep and Conway [19]. Four different dried durian seed gum powder (10 g) were weighed and the dried powder was allowed to flow through the funnel into the base and a pile was formed at the base. The angle of repose was then calculated as follows [19,27]:

(6) $$\text{Angle}\;\text{of}\;\text{repose}\;\left(\text{tanθ}\right)=\frac{H}{R}$$

H is the height of the cone formed after complete flow and R is the radius of the cone used to determine the angle of repose.

#### Solubility

The solubility was determined according to the previous researchers [28] with minor modification. One g of seed gum powder was added to 100 mL of distilled water. Then, the mixture was agitated with mechanical stirring at the room temperature (25 ± 1°C) and elevated temperature (80°C) for 30 min. The gum solution was then centrifuged at 6000 g for 30 min to remove the insoluble material. The supernatant was transferred to disposable Petri dishes and oven dried at 105°C for 24 h until constant weight. The determination of solubility was carried out in triplicate. Therefore, the average of three measurements was considered for further data analysis:

(7) $$\text{Solubility}\;\left(\%\right)=\left(C_{1}/C_{2}\right)\times 100$$

C_1_ = supernatant concentration (mg); C_2_ = initial solution concentration (mg).

#### Water-and oil-holding capacity (WHC and OHC)

Water-holding capacity (WHC) for durian seed gum powder was determined [52] with minor modification. One g of different dried durian seed gum powder was suspended in 10 mL of distilled water, vortexed for 2 min and then centrifuged with a refrigerated centrifuge 3-18 K (Sartorius, Sigma 3–18, Göttingen, Germany) at 3000 g for 30 min. The free water was decanted and the water absorbed by the samples was expressed as grams of water absorbed per 100 g of seed gum. Oil-holding capacity (OHC) was also determined by dispersing 1 g of four different dried durian seed gum powders in 10 mL of refined sunflower oil, and repeated the experiment. It was expressed as grams of oil absorbed per 100 g of seed gum. The measurements were performed in triplicate for each sample. Finally, WHC and OHC were calculated based on the following equations:

(8) $$\text{WHC}=\left(\text{SSW}-\text{SW}\right)/\text{SW}$$

(9) $$\text{OHC}=\left(\text{OSW}-\text{SW}\right)/\text{SW}$$

Where SSW, SW, and OSW are the swollen sample weight, sample weight, and oil-absorbed sample weight, respectively [52].

#### Foam capacity

Foaming capacity was investigated according to the method described by previous researchers [53] with some modifications. In this assessment, 2 g of the durian seed gum was added to the distilled water and the volume was adjusted to 100 mL to prepare the gum solution (2%). Subsequently, pH of the gum solution was adjusted to 6. This experiment was performed at the ambient temperature. Solution of 60 mL was whipped at 15000 rpm for 2 min with a high-speed homogenizer (APV, Crawley, UK). Foaming capacity was expressed as foam expansion immediately after whipping. Foaming capacity was calculated based on the following equation:

(10) $$\text{Foaming}\;\text{capacity}\;\left(\%\right)=\left[\frac{V-V_{0}}{V_{0}}\right]\times 100\%$$

Where V_0_ and V are the volumes immediately before and after whipping, respectively.

#### Scanning Electron Microscopy (SEM) morphology

The morphology of particles was characterized by using scanning electron microscope (SEM) JEOL JSM-6400 SEM (JEOL, Arcade, NY, USA). The volume-averaged geometric diameter (dg) was determined from 1000 particle counts of the SEM images using Image J software (NIH, Center Drive Bethesda, MD, USA).

### Experimental analysis

In this study, the effect of four different drying methods (i.e. oven drying 105°C, vacuum oven drying 60°C, freeze drying and spray drying) on the flow characteristics and functional properties of durian seed gum was investigated. The purified seed gum was considered as a control sample. The control sample was not subjected to those drying processes. A completely randomized design (CRD) was considered to arrange the treatment runs. The drying process was performed in duplicate for each drying method. The data was subjected to one way analysis of variance (ANOVA) to determine the significant (p < 0.05) differences among the drying methods as compared to the control sample. All data analysis was carried out by using Minitab version 15 (Minitab Inc., PA, USA). Fisher multiple comparison test was used to evaluate significant differences (p < 0.05) among the seed gum obtained by four different drying techniques.

## Abbreviations

ANOVA: One way analysis of variance; CRD: Completely randomized design; D: *Durio*; DF: Dietary fiber; FRFs: Fibre-rich fractions; OHC: Oil holding capacity; OSW: Oil-absorbed sample weight; SSW: Swollen sample weight; SW: Sample weight; WHC: Water-holding capacity.

## Competing interest

The authors declared that they have no competing interest.

## Authors’ contributions

BA carried out all the experiments and data analysis. BA also prepared the drafted manuscript, and all authors read, edited and approved the final manuscript.
